# Using easy-to-collect indices to develop and validate models for identifying metabolic syndrome and pre-metabolic syndrome

**DOI:** 10.3389/fendo.2025.1587354

**Published:** 2025-06-11

**Authors:** Chao Shi, Yin Cheng, Ling Ma, Lanqiqi Wu, Hongjuan Shi, Yining Liu, Jinyu Ma, Huitian Tong

**Affiliations:** ^1^ People’s Hospital of Ningxia Hui Autonomous Region, Ningxia Medical University, Yinchuan, Ningxia Hui Autonomous Region, China; ^2^ Ningxia Institute of Clinical Medicine, People’s Hospital of Ningxia Hui Autonomous Region, Yinchuan, Ningxia Hui Autonomous Region, China; ^3^ School of Public Health, Ningxia Medical University, Yinchuan, Ningxia Hui Autonomous Region, China

**Keywords:** metabolic syndrome, pre-metabolic syndrome, easy-to-collect indices, diagnostic model, identifying

## Abstract

**Background:**

This study aimed to develop and validate models for identifying individuals at high risk for metabolic syndrome (MetS) and pre-MetS using easily collectible indices.

**Methods:**

A cross-sectional analysis was conducted using data from the Ningxia Cardiovascular Disorders Survey (NCDS) in China, collected between January 2020 and December 2021. The study population comprised 10,520 participants with complete demographic, anthropometric, and laboratory data. The diagnostic models for MetS were developed using five easily collectible indicators. The performance of the models was compared with that of Lipid Accumulation Product (LAP), Triglyceride-Glucose (TyG) Index, and Waist-to-Height Ratio (WHtR). These same models were subsequently applied to pre-MetS detection as a secondary analysis. Area under the receiver operating characteristic curve (AUC), Hosmer and Lemeshow test, bootstrap method, Brier score and Decision Curve Analysis were employed to evaluate the performance of the models.

**Results:**

Model 1 comprised factors such as WC, SBP, DBP and gender. In contrast, Model 2 included all the variables from Model 1 while additionally incorporating FPG. In the training set, the AUC for Model 1 and Model 2 were 0.914 and 0.924, respectively. The AUC for Model 1 and Model 2 in identifying the presence of pre-MetS and MetS conditions were 0.883 and 0.902, respectively. In the external validation set, the AUC for Model 1 and Model 2 in identifying the presence of MetS were 0.929 and 0.934, respectively. For detecting pre-MetS and MetS conditions, the AUC for Model 1 and Model 2 were 0.885 and 0.902, respectively. Compared to TyG, LAP, and WHtR, model 1 and 2 exhibited a superior ability to identify MetS as well as pre-MetS and MetS conditions in both the training and validation sets.

**Conclusions:**

Our models offered an easy, accurate and efficient tool for identifying MetS and pre-MetS, which might be used in large-scale population screening or self-health management at home.

## Introduction

Metabolic syndrome (MetS) comprises a suite of metabolic markers that significantly increases the risk of developing type 2 diabetes mellitus (T2DM) and cardiovascular diseases (CVD) (1–3). The prevalence of MetS has risen globally over the past few decades (1, 4, 5). Due to its complex and incompletely understood pathogenesis of MetS, early screening in seemingly healthy populations is clinically important for identifying at-risk individuals and enabling timely intervention (6–11).

Since the World Health Organization (WHO) first defined MetS in 1998, multiple diagnostic criteria have emerged. The WHO initially required insulin resistance (IR), impaired glucose tolerance (IGT), or T2DM, as essential components (12). However, in 2001, the National Cholesterol Education Program Adult Treatment Panel III (ATP III) proposed a different set of criteria, later revised by the American Heart Association/National Heart, Lung, and Blood Institute (the revised ATP III) in 2005 (13, 14). This version focused on waist circumference (WC), blood lipids, blood pressure, and fasting plasma glucose (FPG) while removing IR as a mandatory criterion. In 2006, the International Diabetes Federation (IDF) introduced a new standard emphasizing central obesity (15), followed by the Joint Committee for Developing Chinese Guidelines (JCDCG) in 2007, which adapted the criteria for the Chinese population (16).

Despite these developments, MetS diagnosis remains challenging because inconsistent criteria may yield varying results for the same individual. The complexity of these standards also limits their use in large-scale screening or self-health management, particularly among individuals with lower education levels. Consequently, identifying simpler, more accessible screening tools to efficiently assess MetS risk in clinical settings remains a key research priority.

Anthropometric measurements provide a convenient method for detecting MetS and its constituent elements (17–20). Three well-validated indicators have demonstrated particular diagnostic value. Lipid Accumulation Product (LAP) is an innovative metric that offers insights into individuals’ visceral fat content, leveraging both serum triglyceride (TG) levels and WC (21, 22). The Triglyceride-Glucose (TyG) index, composed of TG and FPG, is a novel indicator that has been shown to be correlated with direct markers of IR (23). While LAP and TyG show strong diagnostic performance, their reliance on laboratory testing limits point-of-care application. Waist-to-Height Ratio (WHtR) offers greater accessibility but may lack sufficient sensitivity due to its simplified parameters (24).

To tackle this issue, this study was designed to construct and validate of a diagnostic model incorporating clinical parameters and multiple anthropometric indicators for MetS using data from a large population-based survey of cardiovascular disorders and associated risk factors in Ningxia Hui Autonomous Region, China. This approach enables effective family-based health monitoring, allowing individuals to track their metabolic status in real-time and potentially prevent MetS progression and related complications.

## Methods

### Study population

A cross-sectional investigation was conducted at Ningxia Hui Autonomous Region from January 2020 to December 2021 to examine the prevalence and risk factors associated with various cardiovascular conditions, including hypertension, dyslipidemia, obesity, diabetes mellitus, coronary heart disease, and hyperuricemia. This survey, referred to as the Ningxia Cardiovascular Disorders Survey (NCDS), provided the samples utilized in the present study. Briefly, a district-representative sample was selected from the general population aged 18 and above using a four-phase stratified cluster sampling method. In the initial phase, the nine districts in Ningxia Hui Autonomous Region were classified by economic and administrative tiers. In the next stage, two towns from each county were randomly selected using simple random sampling (SRS) based on a roster provided by local Centers for Disease Control and Prevention. Three communities or villages were chosen from each town during the third stage with SRS. Sampling stratification was performed during the final stage with age and gender distribution based on China census data in 2010. The survey was conducted from January 2020 to December 2021, with a total of 10,803 participants. Before initiating the survey, written informed consent was obtained from all participants, which included consent for both their participation in the study and the use of collected data for future scientific research purposes.

In addition to the inclusion and exclusion criteria of the NCDS, the present study required further exclusions: (a) individuals lacking complete demographic, anthropometric, or laboratory information; (b) individuals with significant outliers in the collected data; (c) those diagnosed with cancer or end-stage renal disease; (d) individuals who have undergone surgical treatment within six months prior to sampling. Finally, our diagnostic models were constructed and validated based on 10520 eligible participants (**Supplementary Figure 1**).

The participants were randomly allocated into two groups—a training set and an external validation set—based on the nine study locations of the NCDS. The training set included 56% of the participants (N=5850) from Jinfeng, Pingluo, Yanchi, and Shapotou to develop the models to identify the presence of MetS from all participants. The external validation set included 44% of the participants (N=4670) from Dawukou, Yongning, Litong, Longde and Xiji to validate the diagnostic performance of these models. **Supplementary Figure 2** illustrates the sampling framework and geographic distribution. The geographic cluster sampling intentionally captured regional diversity, resulting in natural variations between training and validation cohorts that enhance the evaluation of model generalizability.

### Measurements of anthropometric and laboratory data

In the morning, participants had to go to a designated community or village health center after fasting for at least 8 hours. Their anthropometric data, including WC, weight, and height, were measured using standard techniques. After resting for 5 minutes, their right-arm sitting blood pressure was measured three times with an electronic blood pressure monitor (OMRON, HBP-1120U) and then averaged.

Participants’ fasting blood samples (about 5 mL per person) were placed into tubes with sodium heparin as an anticoagulant and spun at 1500 rpm for 10 minutes. The clear liquid on top was then transferred to a small freezer-safe container, kept at -80°C, and shipped to the CIC Medical Laboratory Center in Beijing for testing. The lab used a Beckman Coulter AU5800 machine (from the USA) and reagents from Biosino in Beijing to check levels of LDL-C, FPG, TG, HDL-C, and TC. HbA1c was measured with a Tosoh H-LC-723GX analyzer (from Japan). All tests followed proper guidelines and rules.

### Definitions of MetS and pre-MetS

As per the IDF, MetS was identified by central obesity (WC of at least 90 cm for Chinese men and 80 cm for Chinese women), along with any two of the following four risk factors: (1) High TG of 1.7 mmol/L or more, or being treated for this lipid issue; (2) Low HDL-C, below 1.29 mmol/L for women and below 1.03 mmol/L for men, or receiving treatment for this lipid condition; (3) Blood pressure with systolic (SBP) of 130 mmHg or more, or diastolic (DBP) of 85 mmHg or more, or being treated for previously diagnosed high blood pressure; (4) FPG of 5.6 mmol/L or more, or having a prior diagnosis of T2DM.

In line with this widely accepted research practice (25, 26), pre-MetS refers to individuals who exhibited at least two of the aforementioned five metabolic abnormalities, yet do not fulfill the diagnostic criteria for MetS as set by the IDF.

### Anthropometric indexes calculation

In this study, the diagnostic model would also be compared with LAP, TyG and WHtR regarding their capacity to detect the occurrence of MetS and pre-MetS. The calculations for these three indices are as follows:

LAP (female) = [WC (cm) - 58] × TG (mmol/L);

LAP (male) = [WC (cm) - 65] × TG (mmol/L) (27);

TyG Index = ln[TG (mg/dL)  × FPG (mg/dL)/2] (28);

WHtR = WC (cm)/height (cm) (29).

### Statistical analyses

All statistical analyses were conducted using STATA MP17 (StataCorp, USA). Descriptive statistics summarized the participants’ physical and metabolic traits, with continuous variables presented as mean ± standard deviation (SD) for near-normal distributions and as median for skewed distributions. Normality was assessed using the Kolmogorov-Smirnov test. Differences between the non-MetS and MetS groups were evaluated using the Chi-square test for categorical variables and one-way analysis of variance for continuous variables.

#### Variable selection criteria

Variables were selected based on clinical relevance and established MetS diagnostic criteria. WC, SBP, DBP, and gender were chosen for their fundamental role in MetS definitions, while FPG was included due to its widespread availability. These variables were assessed using univariate logistic regression, and those with *P* < 0.05 were included in the multivariate models.

#### Handling of missing data

Missing data were managed through complete case analysis, including only participants with complete data for all variables. This approach leveraged the large sample size to ensure sufficient statistical power. Sensitivity analysis using multiple imputation confirmed the models’ robustness.

#### Model calibration techniques

Model calibration was assessed using the Hosmer-Lemeshow (H-L) test and calibration curves. The H-L test evaluated the agreement between observed and predicted outcomes (*P* < 0.001 indicating potential calibration errors). Calibration curves visually confirmed that the models were well-calibrated overall. Bootstrap resampling (500 iterations) was used to assess model stability and internal validation.

Diagnostic models for MetS were established using multivariate logistic regression. The cutoff value for diagnosing MetS was determined by the threshold corresponding to the maximum Youden index. Model performance was also evaluated using metrics such as the area under the receiver operating characteristic curve (AUC) and Decision Curve Analysis (DCA).

### Ethics statement

This study strictly adhered to international ethical guidelines, including the Declaration of Helsinki (2013 revision). The study protocol was reviewed and approved by the Institutional Review Board of People’s Hospital of Ningxia Hui Autonomous Region and obtained ethical approval (Approval No. 2020-YC-002) prior to implementation.

## Results

### Characteristics of the study population

**Table 1** displays the characteristics of the participants. This study encompassed a total of 10520 participants, with 5850 (56.00%) in the training set and 4670 (44.00%) in the validation set. While significant differences (*P*<0.05) were observed in some characteristics (age, education, WC, HbA1c, FPG, HDL-C) between training and validation sets, these variations reflect expected geographic and demographic heterogeneity across Ningxia’s regions due to our cluster sampling design. Such differences do not indicate selection bias but rather enhance the robustness of external validation by testing model performance across diverse subpopulations. The participants in the training set demonstrated a higher prevalence of individuals with a high school education or higher, along with elevated levels of WC and FPG, as well as lower HbA1c levels compared to those in the validation set. The comparison of characteristics between MetS and Non-MetS in the training and validation sets is shown in **Supplementary Table 1**.

**Table 1:** Selected characteristics of participants in the training set and validation sets.

Variables	Overall	Training set	External validation set	*P* value ^a^
Total, n (%)	10520 (100.00)	5850 (56.00)	4670 (44.00)	
Age, Median (years)	46 (33,59)	46 (33,59)	46 (34,59)	<0.05^b^
Gender, n (%)				0.145^c^
Male	4841 (46.02)	2655 (45.38)	2186 (46.81)	
Female	5679 (53.98)	3195 (54.62)	2484 (53.19)	
Smoking, n (%)				0.151^c^
No	8002 (76.07)	4419 (75.54)	3584 (76.75)	
Yes	2517 (23.93)	1431 (24.46)	1086 (23.25)	
Drinking, n (%)				0.425^c^
No	8293 (78.83)	4595 (78.55)	3698 (79.19)	
Yes	2227 (21.17)	1255 (21.45)	972 (20.81)	
Education, n (%)				<0.05^c^
Primary school or below	3707 (35.24)	1927 (32.94)	1780 (38.12)	
Middle school	3016 (28.67)	1674 (28.62)	1342 (28.74)	
High school or above	3797 (36.09)	2249 (38.44)	1548 (33.15)	
Anthropometry Data, Mean ± SD				
Height (cm)	162.65 ± 8.74	162.53 ± 8.79	162.80 ± 8.68	0.113^d^
Weight (kg)	66.46 ± 12.58	66.31 ± 12.57	66.64 ± 12.59	0.192^d^
WC (cm)	83.65 ± 11.70	84.16 ± 12.23	83.01 ± 10.96	<0.001^d^
SBP (mmHg)	130.32 ± 19.96	130.05 ± 20.07	130.65 ± 19.82	0.127^d^
DBP (mmHg)	81.03 ± 11.16	80.96 ± 11.44	81.12 ± 10.81	0.453^d^
Laboratory data, Mean ± SD				
HbA1c (%)	5.64 ± 0.78	5.66 ± 0.77	5.61 ± 0.80	<0.05^d^
FPG (mmol/L)	5.71 ± 1.46	5.73 ± 1.44	5.67 ± 1.48	<0.05^d^
TG (mmol/L)	1.50 ± 1.33	1.50 ± 1.39	1.50 ± 1.25	0.796^d^
TC (mmol/L)	4.27 ± 0.97	4.26 ± 1.00	4.29 ± 0.94	0.163^d^
HDL-C (mmol/L)	1.26 ± 0.28	1.28 ± 0.28	1.24 ± 0.27	<0.001^d^
LDL-C (mmol/L)	2.59 ± 0.78	2.58 ± 0.79	2.60 ± 0.75	0.191^d^

WC, waist circumference; SBP, systolic blood pressure; DBP, diastolic blood pressure; HbA1c, glycated hemoglobin; FPG, fasting plasma glucose; TG, triglycerides; TC, total cholesterol; HDL-C, high density lipoprotein cholesterol; LDL-C, low density lipoprotein cholesterol.

^a^ *P* values were calculated to compare the characteristics of the training set and validation set.

^b^ *P* values were obtained from Kolmogorov-Smirnov test.

^c^ *P* values were obtained from chi-square test.

^d^ *P* values were obtained from ANOVA.

### Development of diagnostic models for MetS

**Table 2** shows that univariate logistic regression analysis indicated significant independent impacts of WC, SBP, DBP, gender, and FPG on the occurrence of MetS, with notable correlations (*P*< 0.05). This indicated that each of these factors individually contributes to the likelihood of developing MetS. Referring to MetS as the outcome event, WC, SBP, DBP and gender were integrated into the development of a multivariate logistic regression model, designated as Model 1. Furthermore, WC, SBP, DBP, gender, and FPG were included in the formulation of an additional multivariate logistic regression model to assess the extent to which incorporating FPG enhances the performance of Model 1; this was referred to as Model 2.

**Table 2:** Results of univariate and multivariate logistic regression analysis based on 5850 participants in training set.

Variable	Univariate analysis		Multivariate analysis (Model 1) ^a^		Multivariate analysis (Model 2) ^b^	
	Coefficients (95%CI)	OR (95%CI)	Coefficients (95%CI)	OR (95%CI)	Coefficients (95%CI)	OR (95%CI)
Gender						
Female	Reference	Reference	Reference	Reference	Reference	Reference
Male	-0.207 (-0.317,-0.096)	0.813 (0.728,0.909)	-1.718 (-1.896,-1.539)	0.179 (0.150,0.214)	-1.838 (-2.025,-1.651)	0.159 (0.132,0.192)
WC	0.157 (0.148,0.165)	1.170 (1.160,1.180)	0.181 (0.170,0.191)	1.198 (1.186,1.209)	0.185 (0.173,0.196)	1.203 (1.189,1.217)
SBP	0.050 (0.047,0.054)	1.051 (1.048,1.055)	0.029 (0.023,0.034)	1.025 (1.018,1.031)	0.024 (0.019,0.030)	1.025 (1.019,1.030)
DBP	0.068 (0.062,0.073)	1.070 (1.064,1.076)	0.017 (0.008,0.026)	1.019 (1.009,1.030)	0.020 (0.010,0.030)	1.020 (1.011,1.030)
FPG	0.597 (0.537,0.657)	1.817 (1.710,1.929)	–	–	0.500 (0.429,0.572)	1.649 (1.536,1.772)

WC, waist circumference; SBP, systolic blood pressure; DBP, diastolic blood pressure; FPG, Fasting plasma glucose.

a The model 1 incorporated WC, SBP, DBP and gender, with an intercept of -20.892.

b The model 2 incorporated WC, SBP, DBP, gender and FPG, with an intercept of -23.825.

**Supplementary Table 2** shows that Model 1 has a sensitivity of 93.00% and specificity of 78.00%, with an optimal cutoff value of 0.254. Model 2 has a sensitivity of 91.00% and specificity of 80.00%, with an optimal cutoff value of 0.267. The slight decrease in sensitivity but increase in specificity in Model 2 indicates that adding FPG improves the model’s ability to correctly identify individuals without MetS. Both models had strong predictive capabilities, with Model 2 slightly better in specificity.

### Assessment of the diagnostic performance of the models

**Figure 1:**
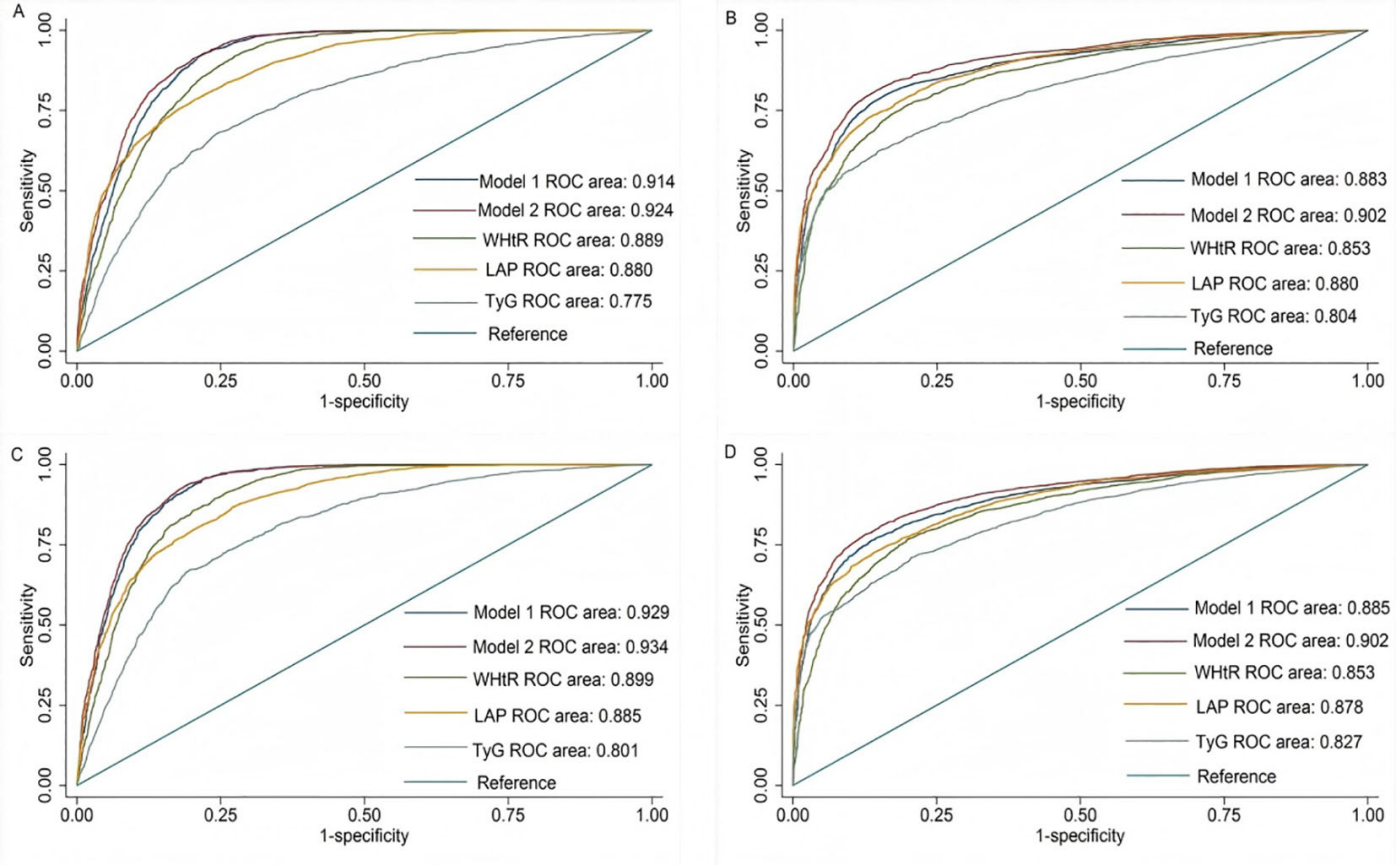
ROC curves of the WHtR, LAP, VAI, TyG index and our model to to identify the presence pre-MetS and MetS. **(A)** ROC curves of the Model1, Model 2, WHtR, LAP, VAI and TyG to identify the presence of MetS in training set. **(B)** ROC curves of the Model1, Model 2, WHtR, LAP, VAI and TyG to identify the presence of pre-MetS and above lesion in training set. **(C)** ROC curves of the Model1, Model 2, WHtR, LAP, VAI and TyG to identify the presence of MetS in validation set. **(D)** ROC curves of the Model1, Model 2, WHtR, LAP, VAI and TyG to identify the presence of pre-MetS and above lesion in validation set. WHtR, waist-to-height ratio, LAP, lipid accumulation product, VAI, visceral adiposity index, TyG index, triglyceride-glucose index.

In the training set, both models demonstrated excellent diagnostic performance for MetS, with Model 1 achieving an AUC of 0.914 (95% CI: 0.907-0.921) and Model 2 reaching 0.924 (95% CI: 0.917-0.930) (**Figure 1A**). For the combined pre-MetS and MetS conditions, the AUC values were 0.883 (95% CI: 0.874-0.892) and 0.902 (95% CI: 0.894-0.910) for Models 1 and 2, respectively (**Figure 1B**). These results were consistently superior to existing indices (all *P*<0.001 for comparisons with TyG, LAP, and WHtR).

The external validation confirmed these findings, with Model 1 (AUC=0.929, 95% CI: 0.918-0.940) and Model 2 (AUC=0.934, 95% CI: 0.924-0.944) maintaining high discriminatory ability for MetS (**Figure 1C**). Similar performance was observed for pre-MetS and MetS detection (Model 1: AUC=0.885, 95% CI: 0.871-0.899; Model 2: AUC=0.902, 95% CI: 0.890-0.914) (**Figure 1D**), with both models again significantly outperforming traditional indices (all *P*<0.001).

These results demonstrate that our models provide more accurate risk stratification than conventional approaches, suggesting substantial clinical and research utility for MetS identification and prevention.

### Evaluation of calibration degree and clinical practicality of the models

**Figure 2:**
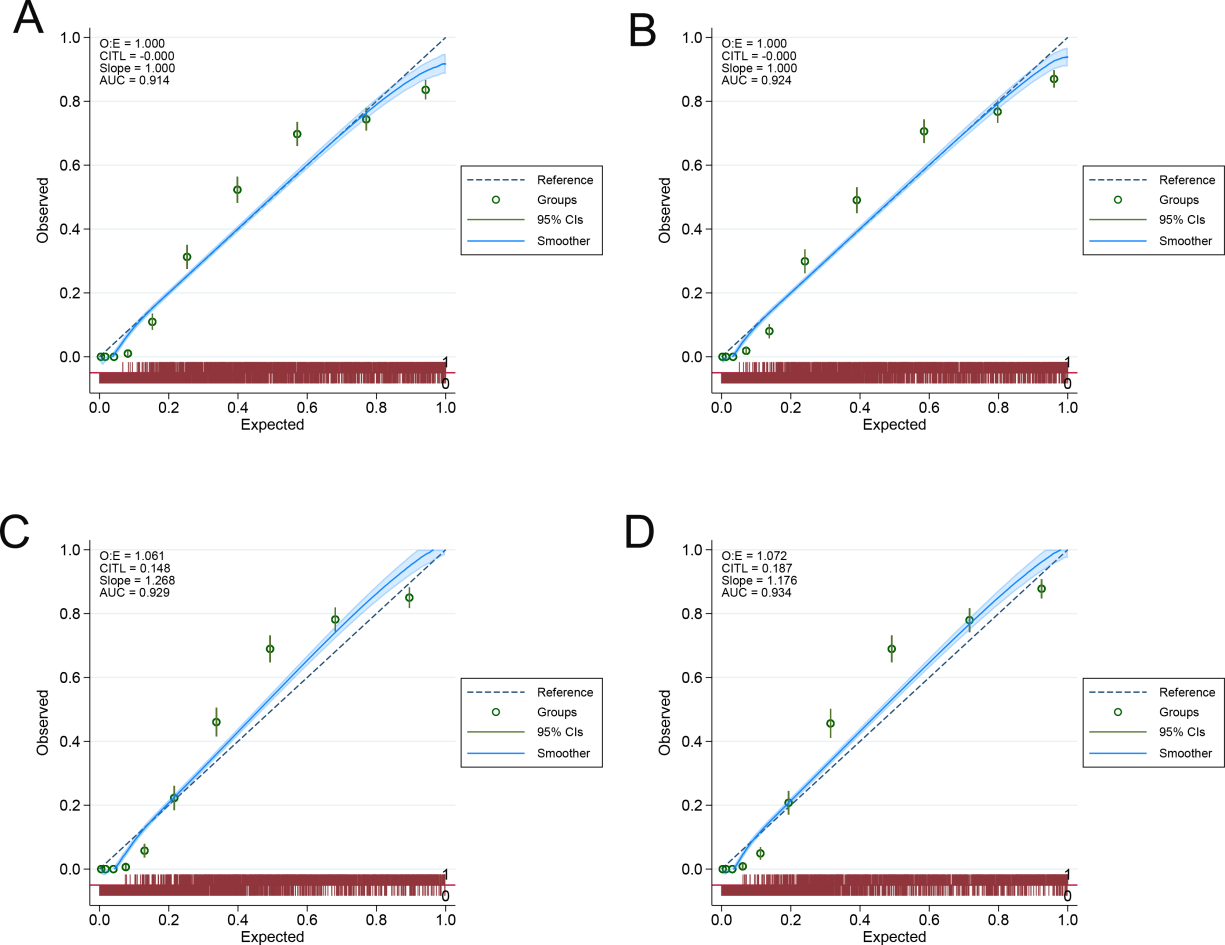
The Hosmer-Lemeshow test for model 1 and model 2 in training and validation sets. **(A, B)** The Hosmer-Lemeshow test for model 1 **(A)** and model 2 **(B)** to identify the presence of MetS in training set. **(C, D)** The Hosmer-Lemeshow test for model 1 **(C)** and model 2 **(D)** to identify the presence of MetS in validation set.

The calibration of the models was comprehensively evaluated using multiple approaches. The H-L test showed statistically significant results (*P*< 0.001) for both models in training and validation sets, which in isolation might suggest calibration issues. However, it is well-documented that the H-L test is particularly sensitive to large sample sizes, where even clinically insignificant deviations can appear statistically significant. The calibration plots (**Figure 2**) demonstrated excellent agreement between predicted and observed probabilities across all risk deciles. Both models showed points closely aligned with the ideal 45-degree line in both training (**Figures 2A, B**) and validation sets (**Figures 2C, D**), with narrow confidence intervals indicating precise calibration. This visual evidence strongly supports good model calibration despite the significant H-L test results.

**Figure 3:**
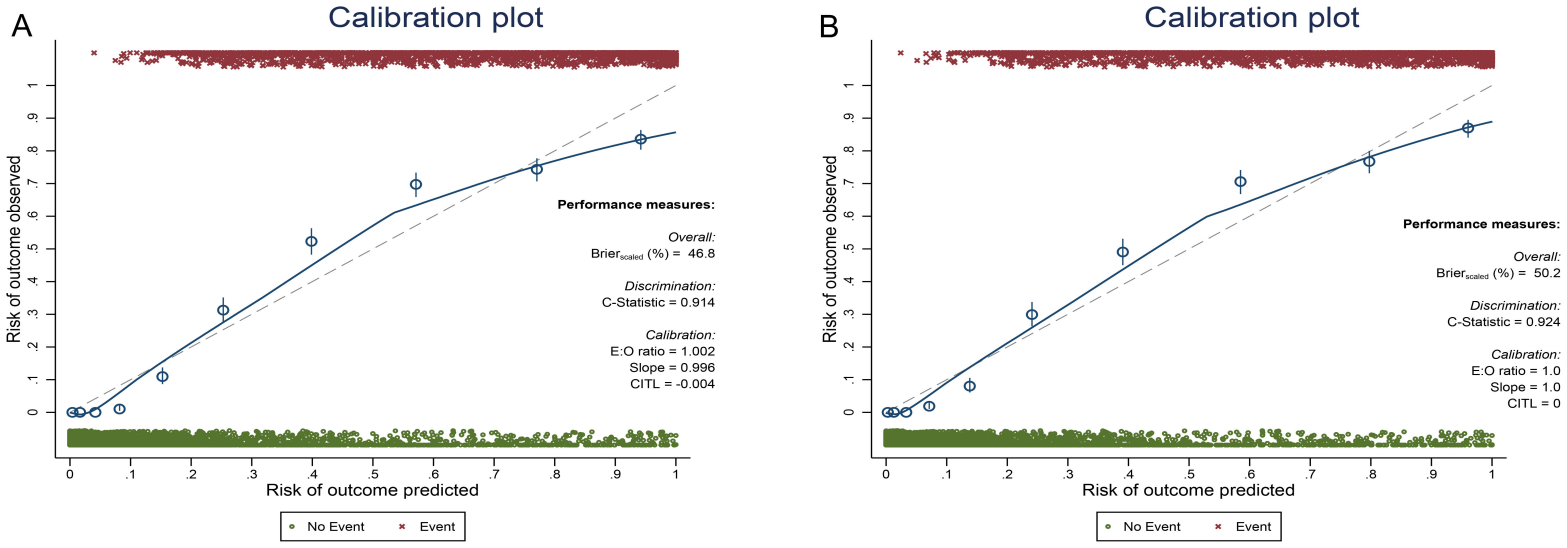
Bootstrap method samples 500 times in the training set for model 1 **(A)** and model 2 **(B)**.

Further supporting the models’ accuracy, bootstrap validation (500 iterations) demonstrated stable performance (**Figure 3**), while Brier scores showed excellent predictive accuracy for both Model 1 (training: 0.116; validation: 0.106) and Model 2 (training: 0.108; validation: 0.102). These scores, all well below the 0.25 threshold indicating useful models, confirm strong calibration. Model 2’s slightly superior performance aligns with its inclusion of fasting plasma glucose. The clinical utility of both models was further validated by Decision Curve Analysis, which demonstrated net benefit across a wide range of probability thresholds in both training (**Supplementary Figures 3A, B**) and validation sets (**Supplementary Figures 3C, D**), supporting their practical application for metabolic syndrome risk assessment.

### Nomogram construction for the models

**Figure 4:**
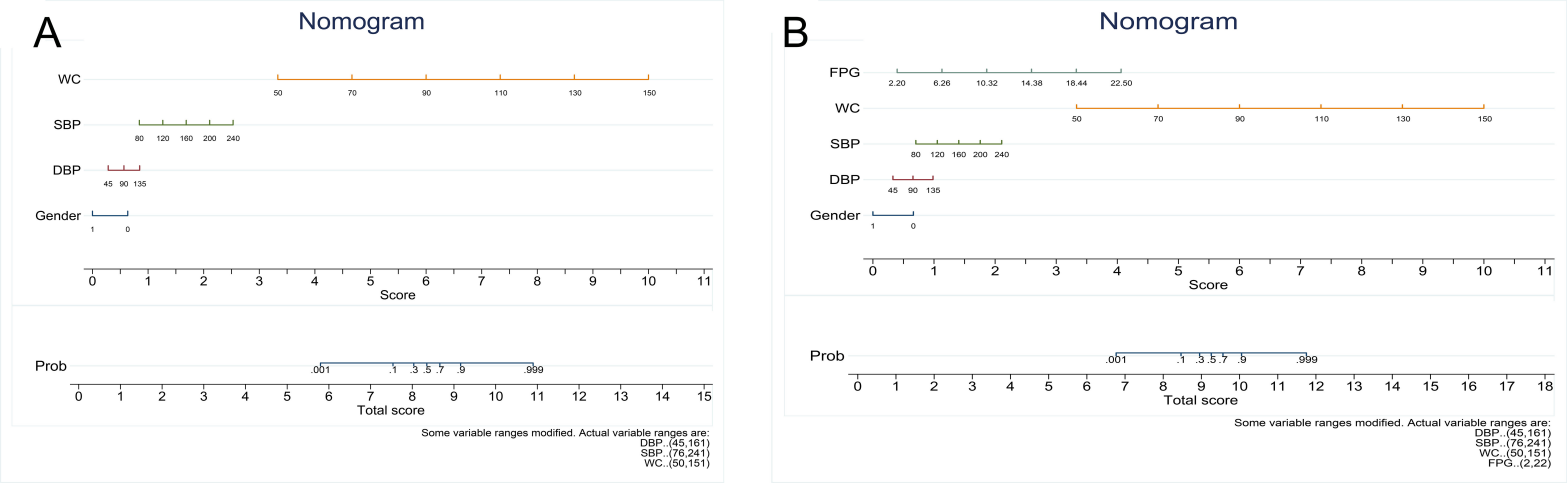
Nomogram model 1 **(A)** and model 2 **(B)** for Metabolic Syndrome. Each indicator in the nomogram prediction model can can get its corresponding score in the integral line in the middleof the graph, and then each corresponding score can be summed up one by one to obtain the total score. which iscorresponding to the probability of the risk of MetS occurrence. WC, waist circumference; SBP, systolic blood pressure; DBP, diastolic blood pressure; FPG, fasting plasma glucose.

To facilitate application, we constructed a nomogram based on two risk prediction models. The scores of independent influencing factors are ascertained based on the sample group data, and the sum corresponding to the prediction probability represents the individual’s MetS risk (**Figure 4**).

## Discussion

In this cross-sectional study, we developed two diagnostic models for MetS using five easily collectible indicators. We then conducted external validation and compared their diagnostic performance with existing indicators, evaluating calibration accuracy and clinical applicability. The models established in this study demonstrated good discrimination, stability, and clinical applicability in identifying the presence of MetS as well as its precursor states, specifically pre-MetS, within the general population. Notably, the diagnostic performance of our models exceeded that of existing metrics such as WHtR, LAP, and TyG, which have been reported to exhibit relatively high diagnostic efficacy in detecting the presence of MetS (18, 20, 24, 30, 31). Our models have been presented in the form of a nomogram, which enables the easy, accurate, and rapid identification of individuals at risk for MetS. These models offer a revolutionize approach to managing MetS by providing innovative frameworks that enable the development and implementation of targeted intervention projects. These projects not only address the complex components of MetS but also empower individuals through self-management strategies, promoting long-term adherence to healthy behaviors and enhancing overall health outcomes.

Disease risk prediction models are primarily used to identify high-risk populations for targeted interventions or self-health management (6). Consequently, simplicity and practicality emerge as critical considerations in the development of these models, particularly among populations with lower educational attainment in resource-limited settings. In this study, our first principle for selecting predictive factors was simplicity, accessibility, and non-invasive. Therefore, we chose four indicators—WC, SBP, DBP and gender that had been included in various definitions of MetS to construct Model 1. At the same time, considering that FPG is an indicator included in various definitions of MetS and has become easily accessible with the widespread use of home blood glucose meters (32), Model 2 was constructed by adding FPG to Model 1. Our findings indicated that both Model 1 and Model 2 demonstrated strong diagnostic efficacy in identifying MetS, with AUC exceeding 90% in both training and validation set. Importantly, the indicators used in the models were easily accessible, thereby enhancing their universality. Besides, the AUC of Model 2 was slightly higher than that of Model 1. However, the choice between these models should also consider the application scenarios and subsequent cost-effectiveness studies.

To develop effective disease prevention strategies, we need models that can identify both healthy individuals and those with pre-MetS or MetS. Our study combined pre-MetS and MetS into one outcome and found that our models performed well, with AUC values over 88% in both training and validation sets. This shows that our models can identify MetS and high-risk populations for early intervention. However, the clinical value of identifying pre-MetS in a cross-sectional study needs more support. While it can guide prevention and reduce the risk of progressing to MetS, our study design doesn’t show how pre-MetS progresses over time. Future longitudinal research is necessary to confirm the models’ predictive accuracy for the transition from pre-MetS to MetS, which would provide a more solid foundation for their clinical application.

Previous studies have highlighted straightforward collection and interpretation, standardized measuring, and non-invasive as the advantages of anthropometric indexes in identifying MetS (24, 33). Our previous study also indicated that among the eight anthropometric and lipid-related indices (BMI, WHtR, weight-adjusted waist index, Conicity index, a body shape index, LAP, visceral obesity index, and TyG index), WHtR, LAP and TyG index were the most effective for identifying the presence of MetS in resource-limited areas (24). Importantly, our current Models 1 and 2 outperformed these individual indices in identifying both MetS and pre-MetS conditions across training and validation datasets. Notably, few previous studies have reported AUC values exceeding 90% for anthropometric-based MetS detection (34), highlighting the superior diagnostic performance of our models. However, the translation to real-world self-screening may be limited by measurement variability in self-administered anthropometrics (particularly WC) and potential calibration differences in consumer-grade monitoring devices.

Although higher WC and FPG levels in the training set could theoretically affect model sensitivity, we observed consistent performance across both datasets. Specifically, the training set had higher mean values of WC (84.16 cm vs. 83.01 cm) and FPG (5.73 mmol/L vs. 5.67 mmol/L) compared to the validation set. Despite these differences, the AUC values for Model 1 and Model 2 in the training set were 0.914 and 0.924, respectively, while in the validation set, they were 0.929 and 0.934, respectively. This indicates that the models performed similarly well in both datasets, suggesting that the differences in WC and FPG did not significantly impact the overall performance.

While the H-L test showed statistically significant results (*P* < 0.001) for both models across training and validation sets, suggesting potential calibration issues, this finding must be interpreted in context. The H-L test groups data by predicted probabilities and compares observed vs. predicted positive rates. Significant differences suggest poor model fit. This method makes results sensitive to sample size changes. Typically, the sample is divided into 10 groups, each with about 10% of the data. Small samples can lead to unreliable results in some groups, while large samples like ours (n=10,520) may cause some groups to have too few data points, affecting test accuracy. In addition, the H-L test statistic is calculated using the average predicted probability in each group, weighted by the number of samples. This calculation method also increases sensitivity to sample size changes (35, 36). More importantly, our bootstrap validation (500 iterations) demonstrated excellent model stability, and the Brier scores confirmed strong predictive accuracy (Model 1: 0.116 training, 0.106 validation; Model 2: 0.108 training, 0.102 validation). These scores, all below the 0.25 threshold indicating useful models, along with calibration curves showing close alignment between predicted and observed probabilities, collectively suggest good calibration despite the H-L test results. The clinical utility was further supported by Decision Curve Analysis, which showed net benefit across wide probability thresholds in both datasets. While we mitigated overfitting through careful predictor selection and external validation, future studies could explore regularization techniques. Together, these findings demonstrate our models’ robust diagnostic performance and clinical applicability for MetS identification.

Last but not least, to broaden the practical utility of our research insights, we have crafted a nomogram derived from the two models. This innovative tool streamlined the process of identifying individuals at risk for MetS, ensuring that the identification was not only straightforward and precise but also swift. By enabling the early detection of MetS, it paved the way for enhanced patient outcomes through the prompt initiation of preventive strategies, including lifestyle adjustments and targeted medical interventions. Moreover, the nomogram’s capacity to support early detection was in line with the increasing focus on preventive healthcare. It facilitates the strategic allocation of resources towards early intervention initiatives, which may markedly alleviate the burden of chronic diseases related to MetS. This approach not only improves individual health outcomes but also contributes to a more efficient and effective healthcare system overall. However, before this tool can be officially promoted and applied in clinical practice, it still needs to undergo application evaluation studies in different regions and among various populations.

### Strengths, limitations and future directions

Our study was characterized by a multitude of strengths. Firstly, according to our knowledge, this was the first diagnostic model for MetS based on the population in Northwest China and one of the largest analyses in this field. Secondly, the samples in this study were drawn from a rigorously designed epidemiological study that the data were collected under a rigorous, standardized protocol. The model development and validation conducted in independent cohorts effectively minimized the influence of overfitting and provided a robust assessment of generalizability, minimizing potential bias in performance evaluation.

Several limitations should be acknowledged. First, our study used a single diagnostic threshold for both MetS and pre-MetS. While this approach simplifies large-scale screening and avoids patient confusion, it may not be optimal for detecting pre-MetS cases. A threshold optimized for MetS may result in lower sensitivity for pre-MetS, potentially leading to missed opportunities for early intervention. This limitation underscores the need for future research to develop and validate separate cutoff values for MetS and pre-MetS to enhance the sensitivity and specificity of diagnostic models. Second, our external validation was conducted solely in Ningxia, which may limit generalizability to other regions with different geographic and socioeconomic characteristics. For instance, variations in urbanization levels, dietary habits, and healthcare infrastructure across regions could influence both metabolic profiles and the accuracy of diagnostic measurements. Future multicenter validation studies across diverse Chinese and international populations are needed to confirm broader applicability. Third, using only IDF criteria may affect generalizability; adopting the harmonized IDF/AHA/NHLBI definition would enhance consistency (37). Besides, the exclusion of lipid profiles (TG and HDL-C), while intentional to prioritize accessibility in resource-limited settings, may limit the model’s ability to detect specific MetS phenotypes, particularly in metabolically obese normal-weight (MONW) individuals. These individuals often exhibit normal BMI but elevated visceral adiposity and dyslipidemia, which are key drivers of metabolic risk. Our models, reliant on WC, blood pressure, and FPG, might underestimate MetS risk in MONW populations where lipid abnormalities are the primary metabolic disturbance. Clinicians should consider supplemental lipid testing when MONW is suspected, even if model scores are normal.

## Conclusions

Our study developed robust diagnostic models that effectively identify both MetS and pre-MetS, demonstrating superior performance to conventional indices (WHtR, LAP, and TyG). The user-friendly nomogram format enables rapid risk assessment in clinical settings. While these findings are promising, future research should: (1) validate the models in multi-ethnic populations using standardized MetS criteria, (2) incorporate longitudinal data to assess predictive validity, and (3) evaluate the added value of lipid biomarkers. Such refinements could establish these tools as versatile solutions for population screening and tailored prevention programs across diverse healthcare settings.

## Data Availability

The data used to support the findings of this study are available from the corresponding author upon request.
