# Supplementary material for: Using easy-to-collect indices to develop and validate models for identifying metabolic syndrome and pre-metabolic syndrome
Source: Front Endocrinol (Lausanne). 2025 Jun 11;16:1587354. doi: 10.3389/fendo.2025.1587354 (PMC12187839; doi:10.3389/fendo.2025.1587354)
Supplement: Supplementary file 1 [file SupplementaryFile1.docx]

| **Supplementary Table 1**. Comparison of selected characteristics between MetS and Non-MetS in training and validation sets | | | | | | | | |
| --- | --- | --- | --- | --- | --- | --- | --- | --- |
| Variables | Training set | | | P value | External validation set | | | P value |
|  | Overall | MetS | Non-MetS |  | Overall | MetS | Non-MetS |  |
| Total, n (%) | 5850 (100.00) | 1891 (32.32) | 3959 (67.68) |  | 4670 (100.00) | 1433 (30.69) | 3237 (69.31) |  |
| Age, Median (years) | 46 (33,59) | 55 (44,68) | 40 (28,54) | <0.001b | 46 (34,59) | 54 (42,65) | 42 (31,56) | <0.001b |
| Gender, n (%) |  |  |  | <0.001c |  |  |  | <0.05c |
| Male | 2655 (45.38) | 793 (41.94) | 1862 (47.03) |  | 2186 (46.81) | 626 (43.68) | 1560 (48.19) |  |
| Female | 3195 (54.62) | 1098 (58.06) | 2097 (52.97) |  | 2484 (53.19) | 807 (56.32) | 1677 (51.81) |  |
| Smoking, n (%) |  |  |  | 0.253c |  |  |  | 0.386c |
| Yes | 1431 (24.46) | 445 (23.53) | 986 (24.91) |  | 1086 (23.25) | 322 (22.18) | 765 (23.63) |  |
| No | 4419 (75.54) | 1446 (76.47) | 2973 (75.09) |  | 3584 (76.75) | 1111 (77.82) | 2472 (76.37) |  |
| Drinking, n (%) |  |  |  | <0.05c |  |  |  | 0.625c |
| Yes | 1255 (21.45) | 368 (19.46) | 887 (22.40) |  | 972 (20.81) | 292 (20.38) | 680 (21.01) |  |
| No | 4595 (78.55) | 1523 (80.54) | 3072 (77.60) |  | 3698 (79.19) | 1141 (79.62) | 2557 (78.99) |  |
| Education, n (%) |  |  |  | <0.001c |  |  |  | <0.001c |
| Primary school  or below | 1927 (32.94) | 879 (46.48) | 1048 (26.47) |  | 1780 (38.12) | 754 (52.62) | 1026 (31.70) |  |
| Middle school | 1674 (28.62) | 574 (30.35) | 1100 (27.78) |  | 1342 (28.74) | 427 (29.80) | 915 (28.27) |  |
| High school  or above | 2249 (38.44) | 438 (23.16) | 1811 (45.74) |  | 1548 (33.15) | 252 (17.59) | 1296 (40.04) |  |
| Anthropometry data, Mean±SD |  |  |  |  |  |  |  |  |
| Height (cm) | 162.53±8.79 | 161.65±9.14 | 162.95±8.59 | <0.001d | 162.80±8.68 | 162.31±9.22 | 163.02±8.42 | <0.001d |
| Weight (kg) | 66.31±12.57 | 72.96±13.05 | 63.14±11.01 | <0.001d | 66.64±12.59 | 74.42±12.66 | 63.19±10.92 | <0.001d |
| WC (cm) | 84.16±12.23 | 94.48±9.10 | 79.23±10.31 | <0.001d | 83.01±10.96 | 92.74±7.51 | 78.71±9.37 | <0.001d |
| SBP | 130.05±20.07 | 142.22±18.64 | 124.25±18.03 | <0.001d | 130.65±19.82 | 142.61±18.39 | 125.36±18.05 | <0.001d |
| DBP | 80.96±11.44 | 86.45±10.71 | 78.34±10.83 | <0.001d | 81.12±10.81 | 86.47±10.77 | 78.76± 9.95 | <0.001d |
| Laboratory data, Mean±SD |  |  |  |  |  |  |  |  |
| HbA1c (%) | 5.66±0.77 | 5.97±0.97 | 5.51±0.61 | <0.001d | 5.61±0.80 | 5.94±0.99 | 5.47±0.64 | <0.001d |
| FPG (mmol/L) | 5.73±1.44 | 6.35±1.82 | 5.44±1.09 | <0.001d | 5.67±1.48 | 6.28±1.92 | 5.40±1.14 | <0.001d |
| TG (mmol/L) | 1.50±1.39 | 2.07±1.68 | 1.23±1.13 | <0.001d | 1.50±1.25 | 2.15±1.59 | 1.22±0.93 | <0.001d |
| TC (mmol/L) | 4.26±1.00 | 4.48±1.11 | 4.16±0.92 | <0.001d | 4.29±0.94 | 4.54±0.97 | 4.18±0.91 | <0.001d |
| HDL-C (mmol/L) | 1.28±0.28 | 1.18±0.24 | 1.33±0.28 | <0.001d | 1.24±0.27 | 1.13±0.23 | 1.28±0.27 | <0.001d |
| LDL-C (mmol/L) | 2.58±0.79 | 2.75±0.84 | 2.50±0.76 | <0.001d | 2.60±0.75 | 2.79±0.75 | 2.52±0.74 | <0.001d |
| Abbreviations: WC, waist circumference; SBP, systolic blood pressure; DBP, diastolic blood pressure; HbA1c, glycated haemoglobin; FPG, fasting plasma glucose; TG, triglycerides; TC, total cholesterol; HDL-C, high density lipoprotein cholesterol; LDL-C, low density lipoprotein cholesterol; | | | | | | | | |
| a P values were caculaed to compare the characteristics of the training set and validation set. | | | | | | | | |
| b P values were obtained from Kolmogorov-Smirnov test. | | | | | | | | |
| c P values were obtained from chi-square test. | | | | | | | | |
| d P values were obtained from ANOVA. | | | | | | | | |

| **Supplementary Table 2.** Optimal cutoff values for diagnosing metabolic syndrome and pre-metabolic syndrome of the models | | | | | | | | |
| --- | --- | --- | --- | --- | --- | --- | --- | --- |
| Model | Mets | | | | Pre-MetS | | | |
|  | Cutoff | Youden  index | Sensitivity (%) | Specificity (%) | Cutoff | Youden index | Sensitivity (%) | Specificity (%) |
| Model 1a | 0.254 | 0.71 | 93.00 | 78.00 | 0.148 | 0.64 | 80.00 | 84.00 |
| Model 2b | 0.267 | 0.71 | 91.00 | 80.00 | 0.123 | 0.67 | 82.00 | 85.00 |
| Abbreviations: MetS, metabolic syndrome; WC, waist circumference; SBP, systolic blood pressure; DBP, diastolic blood pressure; FPG, Fasting plasma glucose. | | | | | | | | |
| a The model 1 incorporated WC, SBP, and DBP. | | | | | | | | |
| b The model 2 incorporated WC, SBP, DBP, gender and FPG. | | | | | | | | |

| **Supplementary Table 3.** Brier scores for the models in training and validation set | | |
| --- | --- | --- |
| Model | Brier scores, Mean±SD | |
|  | Traing set | Validation set |
| Model 1 ^a^ | 0.116±0.196 | 0.106±0.178 |
| Model 2 ^b^ | 0.108±0.194 | 0.102±0.184 |
| Abbreviations: WC, waist circumference; SBP, systolic blood pressure; DBP, diastolic blood pressure; FPG, Fasting plasma glucose. | | |
| ^a^ The model 1 incorporated WC, SBP, and DBP. | | |
| ^b^ The model 2 incorporated WC, SBP, DBP, gender and FPG. | | |


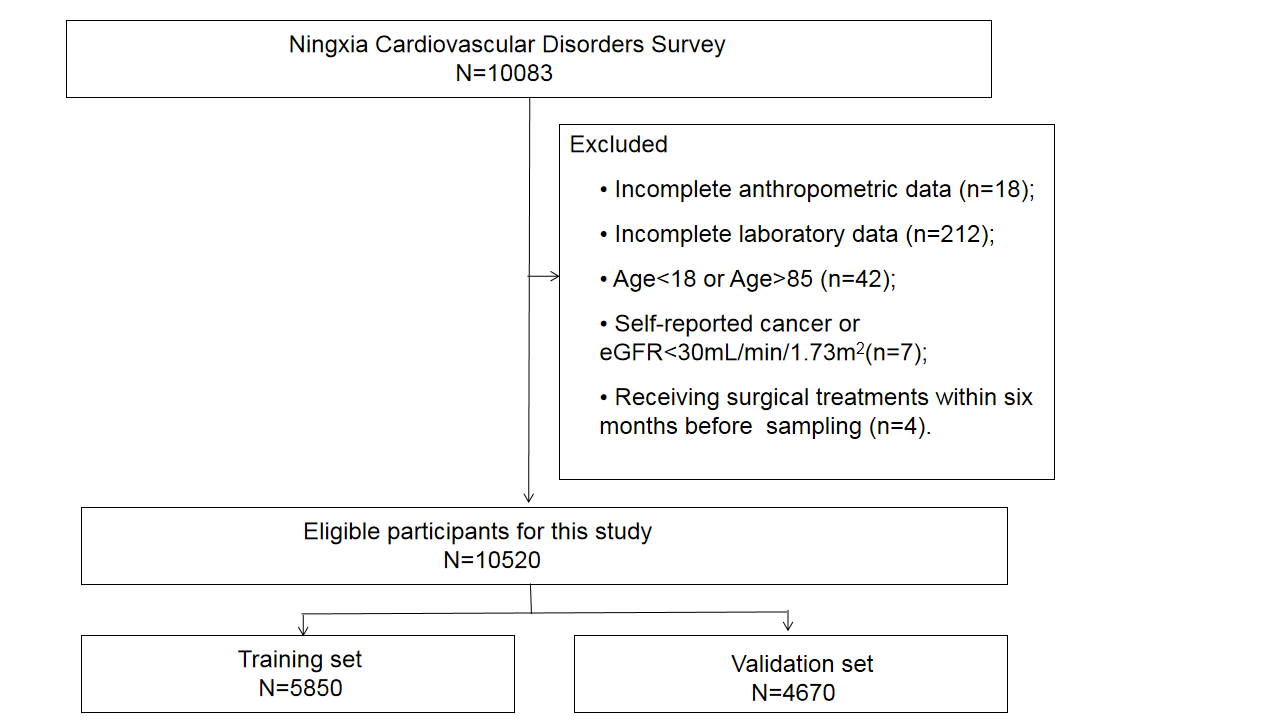


**Supplementary Figure 1.** The flowchart of participant selection for this study.


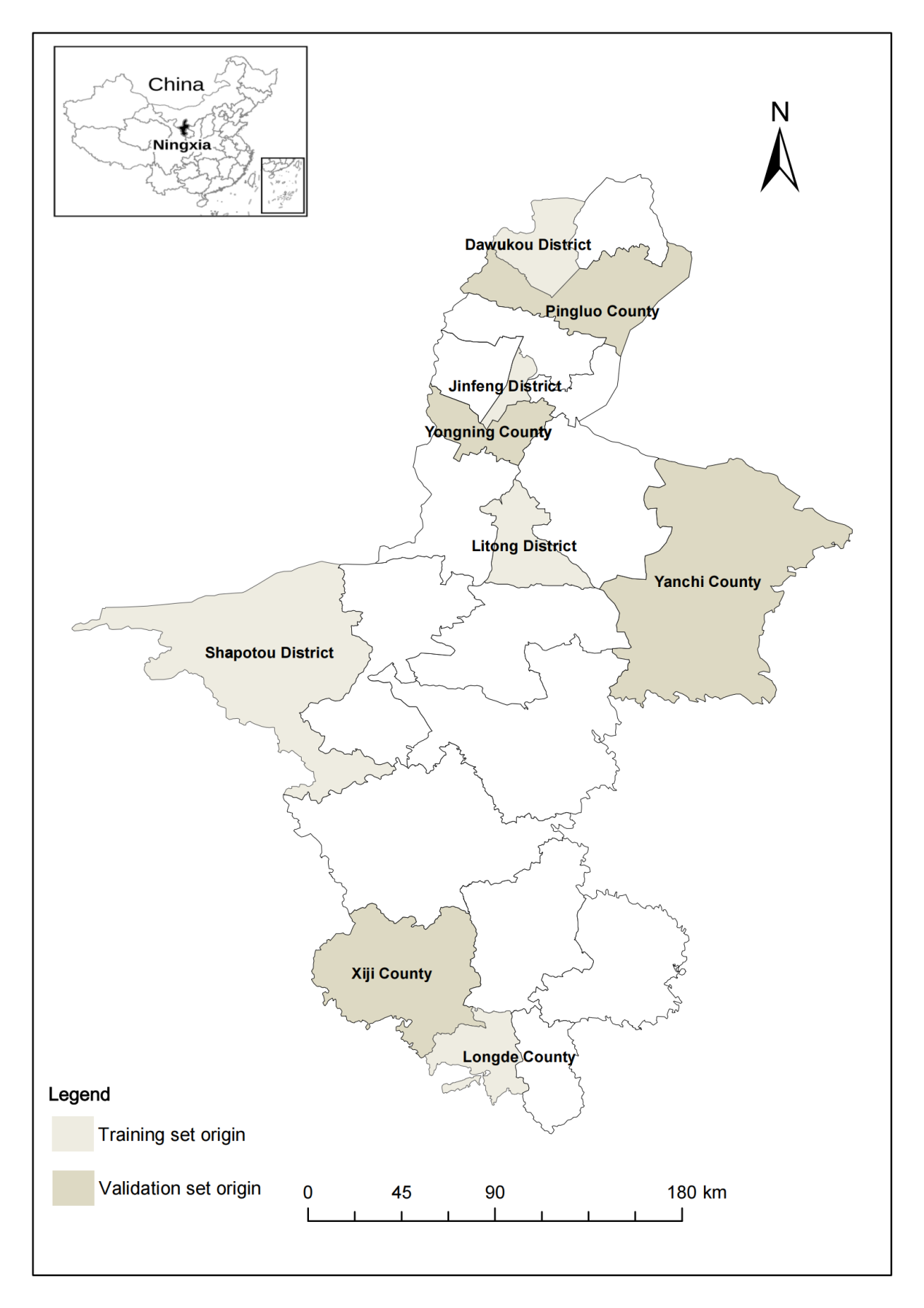


**Supplementary Figure 2.** Sampling regions for training and validation sets.


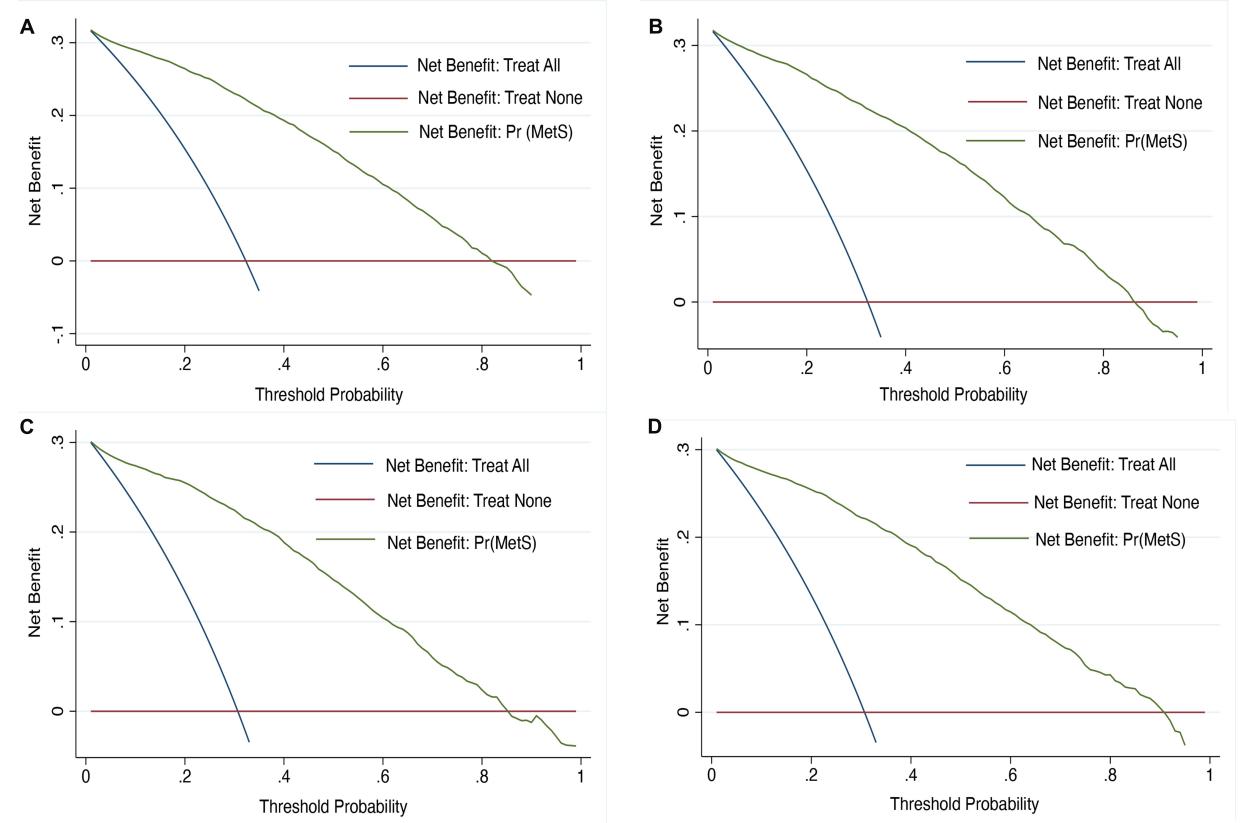


**Supplementary Figure 3.** Decision curve for model 1 and model 2 in training and validation sets. **A-B**. Decision curve for model 1 (A) and model 2 (B) in training set; **C-D**. Decision curve for model 1 (C) and model 2 (D) in validation set.
